# Cultural moderation of emotion regulation strategies: childhood maltreatment and suicidal ideation in Chinese female students

**DOI:** 10.3389/fpsyg.2025.1743177

**Published:** 2026-02-11

**Authors:** Zhaoxia Pan, Dajun Zhang, Xiaohua Bian

**Affiliations:** 1 School of Educational Science, Zhengzhou Normal University, Zhengzhou, China; 2 Faculty of Psychology, Research Center for Mental Health Education, Southwest University, Chongqing, China

**Keywords:** affective balance, childhood maltreatment, Chinese female college students, cultural moderation, cultural psychology, expressive suppression

## Abstract

**Introduction:**

This study examines the complex interplay between childhood maltreatment and suicidal ideation among Chinese female college students within China’s collectivistic context, examining affective balance as a mediator and expressive suppression as a culturally contingent moderator.

**Methods:**

A cross-sectional design was employed with 2,280 female undergraduates from 11 universities across eight Chinese provinces. Participants completed validated measures, including the Childhood Trauma Questionnaire, Beck Scale for Suicide Ideation, Bradburn Affect Balance Scale, and Emotion Regulation Questionnaire. The data were analyzed by conducting moderated mediation analysis with bootstrapping procedures to test the hypothesized pathways.

**Results:**

Affective balance significantly mediated the relationship between childhood maltreatment and suicidal ideation (indirect effect = 0.12, 95% CI [0.08, 0.16]). Expressive suppression moderated this mediation pathway, with higher levels of suppression attenuating the adverse effects of childhood maltreatment on affective balance (interaction effect = −0.09, *p* < 0.01). The protective buffering effect was particularly pronounced at moderate to low levels of childhood trauma.

**Discussion:**

Consistent with prior cross-cultural psychology research, the present findings substantiate the adaptive role of expressive suppression in China’s collectivistic cultural context. Rather than challenging Western emotion regulation models, this study extends the existing evidence by demonstrating context-specific patterns of emotion regulation among Chinese participants. These results underscore the importance of incorporating cultural variability into models of emotion regulation and suggest that culturally informed approaches are essential for understanding emotional adjustment and mental health across societies.

## 1 Introduction

### 1.1 Global burden of youth suicide and cultural variations in risk pathways

Youth suicide remains a significant global public health challenge and a leading cause of death among individuals aged 15–29 years. Hundreds of thousands of young people die by suicide annually, with most deaths occurring in low- and middle-income countries. While Western research typically attributes youth suicide to psychological factors such as emotional dysregulation, hopelessness, or impaired self-control (Brausch et al., 2025; da Silva et al., 2024; Martin et al., 2023), emerging evidence suggests that cultural factors fundamentally shape these risk pathways. In collectivistic societies like China, emotional control and relational harmony are highly valued. Consequently, emotion regulation strategies such as expressive suppression—often viewed as maladaptive in Western psychology—may serve socially adaptive or protective functions within these cultural contexts (Sun and Lau, 2018; José et al., 2011). This divergence highlights how the same emotional behavior can yield different psychological outcomes across cultures.

Chinese female college students represent a particularly vulnerable and relevant group for examining these cultural dynamics. These students face intersecting pressures, including intense academic competition shaped by the examination-oriented educational system, traditional gender role expectations emphasizing emotional restraint, and significant mental health service gaps on university campuses (Qian, 2004). These combined factors make this population especially susceptible to the psychological consequences of emotion regulation strategies, like expressive suppression. Against this Chinese cultural backdrop, societal expectations to maintain composure and suppress distress can create tensions between cultural conformity and personal wellbeing. While suppression may reduce interpersonal conflict and preserve social harmony, prolonged emotional inhibition can limit emotional release and exacerbate internalized distress. Therefore, understanding suicide risk among Chinese youth requires examining how emotion regulation processes are embedded within cultural frameworks.

Accordingly, this study adopts a culturally informed perspective to explore how culturally endorsed emotion regulation strategies influence the emotional pathways linking childhood maltreatment to suicidal ideation. In addition, cultural models emphasize that emotion regulation can serve dialectical goals (i.e., tolerating mixed affect and maintaining balance) and interpersonal goals (i.e., supporting socially engaging emotions that sustain relational harmony) in East Asian contexts (Mauss and Butler, 2010; Kitayama et al., 2006). By doing so, it aims to provide a more contextually grounded understanding of youth suicide in non-Western settings.

### 1.2 Childhood maltreatment: a universal risk factor with cultural modulation

Childhood maltreatment, which encompasses physical, emotional, and sexual abuse along with neglect and economic exploitation, is a well-established transdiagnostic risk factor for suicidal ideation across diverse populations (Bernstein et al., 1997; Angelakis et al., 2019). The cumulative contextual risk model (Yuan and Wang, 2020) offers a theoretical framework suggesting that early adverse experiences escalate vulnerability to psychopathology through developmental cascades, and adult women with a history of childhood maltreatment are at a significant risk of suicidal ideation, as numerous empirical studies have confirmed (Angelakis et al., 2019; Yang et al., 2019; Hoertel et al., 2015). Nevertheless, emerging evidence indicates that cultural norms and values may significantly buffer or exacerbate these effects, thereby creating divergent developmental trajectories across cultural contexts.

Within China’s collectivistic cultural framework, in which the suppression of negative emotional expressions is socially validated and often rewarded (José et al., 2011), individuals with a history of maltreatment may employ expressive suppression as a culturally sanctioned coping strategy, potentially altering established pathways to suicidality. This observation challenges conventional Western psychological perspectives that uniformly categorize emotional suppression as maladaptive (Gross and John, 2003) and aligns with the cultural fit hypothesis (Friedman et al., 2010)—a theoretical framework in cross-cultural psychology positing that emotion regulation strategies are psychologically adaptive when they align with prevailing cultural values, norms, and social expectations (Butler et al., 2007). According to this hypothesis, the psychological consequences of emotion regulation strategies depend not only on their intrinsic properties but also on their cultural context and meaning.

### 1.3 Affective balance as a mediator: integrating cultural and emotional mechanisms

The dual-process model of affect (Fiori, 2009; Diener et al., 2018) conceptualizes wellbeing and psychopathology as arising from the dynamic interplay between positive and negative affective systems. Thus, affective balance—the relative predominance of positive over negative emotions—is a critical indicator of emotional adjustment and a proximal determinant of mental health. When this equilibrium tilts toward negative affectivity, individuals experience pervasive emotional distress, heightened cognitive rumination, and a reduced capacity for adaptive coping, all of which substantially increase their vulnerability to suicidal ideation (Pan et al., 2024). Empirical evidence has consistently demonstrated that lower affective balance is associated with depressive symptomatology and suicidal thoughts across cultural contexts (Humphreys et al., 2020; Veilleux et al., 2020; Chen et al., 2021).

Childhood maltreatment disrupts this affective equilibrium by altering emotional appraisal and stress responses and fostering negativity biases in both attention and memory processing (Alley et al., 2026; Puetz et al., 2020). Individuals exposed to early adverse experiences often exhibit diminished positive affectivity and prolonged negative affective states, reflecting impaired emotional modulation capacity (Goncharenko et al., 2021). Over time, these affective distortions consolidate into a chronic emotional imbalance, which can function as a psychological mechanism—a mediating pathway that links maltreatment to suicidal ideation. In other words, childhood trauma may elevate suicide risk not only through direct psychological distress but also via disrupted affective regulation that undermines emotional homeostasis.

The magnitude and manifestation of this affective disruption vary across cultures. In Chinese collectivistic environments, where emotional restraint and relational harmony are socially reinforced (Cheng et al., 2009), suppression of emotional expression is normally expected; it is frequently associated with social appropriateness rather than emotional dysfunction. Thus, to some extent, expressive suppression may stabilize affective experiences by preventing overt conflict and preserving interpersonal harmony (José et al., 2011). This culturally sanctioned regulatory pattern could potentially mitigate the negative affective consequences of early maltreatment and moderate the strength of the pathway from childhood adversity to suicidal ideation.

Accordingly, this study proposes that affective balance operates as a core emotional mediator linking childhood maltreatment to suicidal ideation, with its mediating strength potentially shaped by the cultural norms surrounding emotion regulation.

Hypothesis 1: Affective balance mediates the association between childhood maltreatment and suicidal ideation in Chinese female college students.

### 1.4 Expressive suppression as a cultural moderator: contextualizing emotion regulation

Expressive suppression, conceptualized as the conscious inhibition of outward emotional expression, is conventionally characterized as maladaptive within Western psychological frameworks owing to its association with heightened physiological arousal, social detachment, and reduced wellbeing (Gross and John, 2003). However, emerging cross-cultural evidence challenges the universality of this claim, suggesting that the adaptiveness of suppression may vary across sociocultural contexts (Butler et al., 2007).

Within collectivistic cultures, such as China, suppression is socially reinforced as an indicator of maturity, self-control, and social appropriateness, particularly among women, whose gender-role socialization emphasizes emotional moderation and relational harmony (Le and Impett, 2013). This observation motivated the present study’s theoretical innovation, namely, the Culturally Moderated Function Hypothesis of Expressive Suppression, which proposes that the psychological function of expressive suppression is culturally contingent. In particular, while suppression tends to undermine adjustment in individualistic societies that prioritize authenticity and expressiveness, it may serve adaptive socio-emotional functions, such as maintaining interpersonal harmony and relational stability, within collectivistic contexts that value restraint and interdependence.

Accordingly, we propose that expressive suppression buffers the affective consequences of childhood maltreatment by stabilizing emotional experiences within culturally sanctioned norms. This reasoning leads to the following hypotheses:

Hypothesis 2 (culturally moderated function hypothesis of expressive suppression): Expressive suppression moderates the indirect effect of childhood maltreatment on suicidal ideation through affective balance such that suppression functions as a protective factor in collectivistic cultural contexts, attenuating the emotional imbalance that links maltreatment to suicidality.

### 1.5 Conceptual model

**Figure 1:**
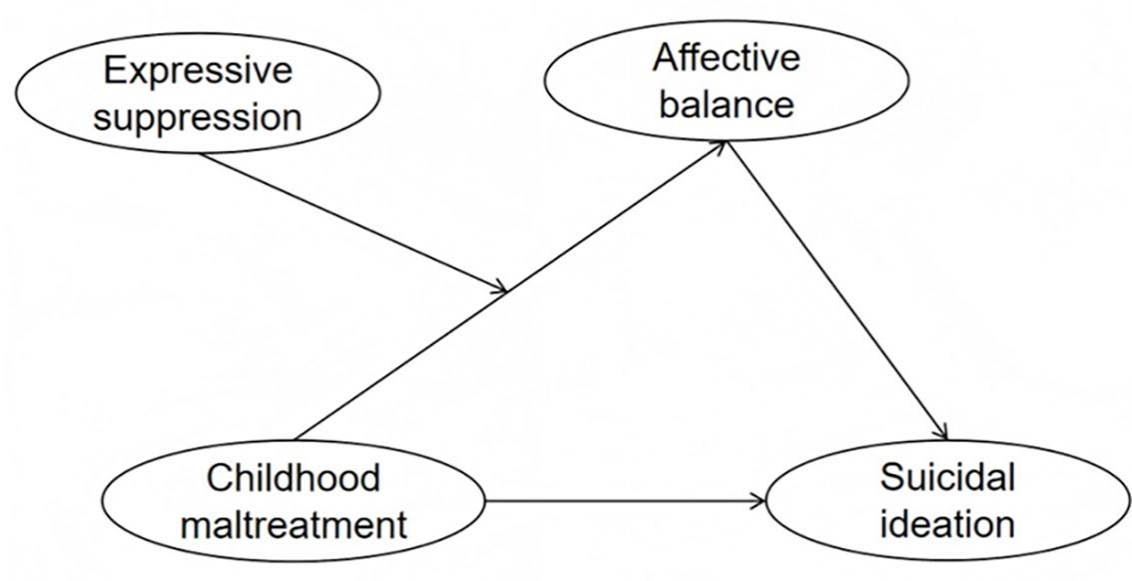
Conceptual model of the hypothesized relationships.

To visually clarify the relationships among childhood maltreatment, affective balance, expressive suppression, and suicidal ideation proposed in this study, Figure 1 presents the moderated mediation conceptual model. In this framework, childhood maltreatment is hypothesized to affect suicidal ideation both directly and indirectly via affective balance (mediation effect; Hypothesis 1). Expressive suppression is theorized to moderate the pathway from childhood maltreatment to affective balance (moderated mediation; Hypothesis 2), reflecting a culturally contingent buffering effect. This integrated model visually illustrates the core hypothesized mechanisms and their interrelations within a collectivistic cultural context.

### 1.6 Research objectives and theoretical innovation

Existing investigations have typically examined environmental risk factors (e.g., childhood maltreatment) and psychological mechanisms (e.g., emotion regulation) in isolation, neglecting their dynamic cultural interplay. This study advances the field in three ways:

1. By testing a comprehensive moderated mediation model that captures culture-specific pathways to suicidality among Chinese female college students.
2. By proposing and empirically testing the Culturally Moderated Function Hypothesis of Expressive Suppression, this novel integrative framework postulates that suppression serves as a culturally contingent regulator that transforms risk into resilience in a collectivistic context.
3. By offering a theoretical revision of Gross and John’s (2003) process model of emotion regulation and reconceptualizing suppression as context-dependent rather than universally maladaptive.

By addressing these conceptual and empirical gaps, this study contributes to global mental health research by demonstrating how cultural variability necessitates theoretical adaptation, rather than the universal application of Western psychological models.

## 2 Method

### 2.1 Participants

A multi-stage cluster sampling approach was employed. First, 11 universities were recruited through convenience collaboration across eight provinces/municipalities in China (Henan, Ningxia, Hainan, Liaoning, Inner Mongolia, Hubei, Jilin, and Beijing). Second, within each participating university, undergraduate classes were treated as the primary sampling units and were randomly selected. Specifically, the research team obtained a complete list of all undergraduate classes from the academic affairs office to establish the sampling frame. From this roster, classes were selected by computer-generated simple random sampling without replacement, with each class assigned a unique identifier and selected with equal probability.

The survey targeted female undergraduate students enrolled in the selected classes. All female students in the chosen classes were invited to participate; no exclusion criteria were applied other than participants having to be female and currently enrolled as an undergraduate. A total of 2,500 questionnaires were distributed to eligible students, and 2,280 valid responses were returned, yielding a response rate of 91.2%. The participants were female college students aged 16–24 years (*M* = 19.87, SD = 1.08).

The distribution across academic years was as follows: first year (28.7%), second year (32.4%), third year (24.8%), and fourth year (14.1%). Concerning region of origin, 32.3% were from eastern China, 41.2% from central China, and 26.55% from western China, which approximately mirrors the national college enrollment distribution. All participants voluntarily took part in the survey and received RMB 5 as compensation upon completion. The study protocol was approved by the Ethics Committee of Zhengzhou Normal University. All procedures adhered to the ethical standards of the 1964 Declaration of Helsinki and its subsequent amendments.

### 2.2 Measures

#### 2.2.1 Maltreatment during childhood

The Chinese version of the Childhood Trauma Questionnaire (CTQ-CF) has been used to assess childhood maltreatment (Zhao et al., 2005). The questionnaire was translated and validated based on the original Childhood Trauma Questionnaire developed by Bernstein et al. (1997). The questionnaire contained 28 items, which were distributed across five dimensions: emotional abuse, physical abuse, sexual abuse, emotional neglect, and physical neglect. Each item was rated by the participants on a 5-point Likert scale ranging from 1 (never) to 5 (always). The subscales ranged from 5 to 25, and the overall score ranged from 25 to 125. Three additional items functioned as validity checks. Higher total scores indicated greater severity of childhood maltreatment. In this study, the overall scale had a Cronbach’s alpha of 0.88, and the Cronbach’s alpha coefficients of the subscales ranged from 0.71 to 0.85.

#### 2.2.2 Suicidal ideation

The Chinese version of the Beck Scale for Suicide Ideation (BSI-CV) was used to assess suicidal ideation (Beck et al., 1961). For Chinese college students, a particular scale has been validated (Li et al., 2010). This scale comprises 19 items rated on a 3-point scale (0–2), assessing the severity of suicidal ideation both during the past week and at the individual’s most depressed or suicidal period. The scale has two components: the first five items assess suicidal thoughts, and the remaining 14 items assess suicidal intentions. In this study, only the first five items associated with suicidal thoughts were used to assess the severity of suicidal ideation during the participants’ most suicidal times, with scores ranging from 0 to 10. Higher scores indicate more intense suicidal ideation and greater suicide risk. Cronbach’s alpha coefficient for the subscale related to suicidal thoughts was 0.83 in this study.

#### 2.2.3 Affective balance

The Chinese version of the Bradburn Affect Balance Scale was used to measure affective balance (Bradburn and Noll, 1969; Wan et al., 2017). The scale includes two dimensions—positive and negative emotions—that are used to determine affective balance. It comprises 10 items in total, each scored as “yes” (1) or “no” (0). In this study, positive emotions were assessed using Items 1, 3, 5, 7, and 9, while negative emotions were assessed using Items 2, 4, 6, 8, and 10. The affective balance score was obtained by subtracting the negative emotion score from the positive emotion score and adding 5 (a constant), yielding a range from 0 to 10. Higher scores indicate greater affective balance. Cronbach’s alpha coefficient for the positive affect subscale in this study was 0.72, and that for the negative affect subscale was 0.69. The overall scale reliability was 0.61, somewhat below the conventional threshold of 0.70, which is consistent with prior research using this measure in Chinese samples (Wan et al., 2017). Despite its modest reliability, the Bradburn Affect Balance Scale was selected for several reasons. First, it has been widely used in cross-cultural research on emotional wellbeing and provides a parsimonious measure of affective balance that aligns with the dual-process model of affect (Diener et al., 2018). Second, its brevity (10 items) reduces participant burden in a comprehensive survey battery. Third, alternative measures, such as the Positive and Negative Affect Schedule (PANAS; Watson et al., 1988), were considered; however, the Bradburn scale was preferred because it directly operationalizes the construct of affective balance (positive minus negative affect) rather than treating positive and negative affect as separate dimensions. This conceptualization better aligns with our theoretical focus on emotional equilibrium as a mediating mechanism. Moreover, prior validation research in Chinese populations has demonstrated adequate construct validity for the Bradburn scale despite its lower internal consistency (Wan et al., 2017).

#### 2.2.4 Expressive suppression

The Chinese version of the Emotion Regulation Questionnaire (ERQ) was used to assess expressive suppression, particularly the expressive suppression subscale. The ERQ was originally developed by Gross and John (2003) and subsequently translated and adapted for the Chinese population by Wang et al., 2007, with demonstrated reliability and validity. The expressive suppression subscale comprises 4 items rated on a 7-point Likert scale, with 1 representing “strongly disagree” and 7 representing “strongly agree.” Scores range from 4 to 28, with higher scores indicating more frequent use of expressive suppression as an emotion regulation strategy. In this study, the Cronbach’s alpha coefficient for the subscale was 0.71, indicating acceptable internal consistency.

### 2.3 Procedure

After receiving approval from university administrators, the researchers collaborated with course instructors to distribute the questionnaires during regular class periods. Before completing the questionnaires, participants were informed of the study’s purpose, assured of confidentiality, and provided written informed consent. A standardized order was followed for administering the questionnaires, starting with demographic information, followed by the CTQ-CF, Affect Balance Scale, ERQ, and BSI-CV. The participants required approximately 25–30 min to complete the questionnaires, and any questions were addressed by research assistants. To ensure confidentiality, completed questionnaires were placed in sealed envelopes.

### 2.4 Data analysis

SPSS 23.0 and PROCESS macro 3.5 were used for data analysis (Hayes, 2018). Descriptive statistics, reliability evaluations, and Pearson correlation tests were performed to investigate the associations among the study variables. Before examining the mediation and moderation effects, Harman’s single-factor test was conducted to evaluate common method bias. Variance inflation factor values were used to check for multicollinearity.

PROCESS macro (Model 4) was utilized with 5,000 bootstrap samples and 95% bias-corrected confidence intervals to test the mediating effect of affective balance (Hypothesis 1). The confidence interval was examined to determine whether it contained zero, which indicates the significance of the indirect effect. In the moderated mediation analysis (Hypothesis 2), PROCESS macro (Model 7) was used with the same bootstrap and confidence interval settings. Before these analyses, all continuous variables were standardized (*z*-score transformation) to facilitate the interpretation of interaction effects. Simple slope analyses were performed to break down the significant interaction effects, which explored the relationship between childhood maltreatment and affective balance at high (+1 SD) and low (−1 SD) levels of expressive suppression.

## 3 Results

### 3.1 Assessment of common method bias

To address potential common method bias, we conducted Harman’s single-factor test using principal-axis factoring. The analysis yielded eight factors with eigenvalues greater than 1, collectively accounting for 58.45% of the variance. The first factor explained 19.18% of the variance, below the critical threshold of 40%. These findings suggest that common method bias did not significantly influence the results of this study.

### 3.2 Description and correlation statistics

Table 1 presents the means, standard deviations, and bivariate correlations of the study variables. Childhood maltreatment was positively correlated with expressive suppression (*r* = 0.06, *p* < 0.01) and suicidal ideation (*r* = 0.34, *p* < 0.01) and negatively correlated with affective balance (*r* = −0.19, *p* < 0.01). Neither affective balance nor suicidal ideation was significantly correlated with expressive suppression. Affective balance was negatively correlated with suicidal ideation (*r* = −0.28, *p* < 0.01).

**Table 1:** Means, standard deviations, and correlations among the study variables.

Variables	*M ±* SD	1	2	3	4
1. Grade	2.34 ± 1.10	1			
2. Childhood maltreatment	34.15 ± 11.70	0.097**	1		
3. Expressive suppression	16.71 ± 4.72	−0.003	0.061**	1	
4. Affective balance	6.36 ± 2.01	−0.047*	−0.186**	−0.016	1
5. Suicidal ideation	7.07 ± 2.56	0.032	0.340**	0.038	−0.284**

*N* = 2,280. *p < 0.05. **p < 0.01.

### 3.3 Mediating effect of affective balance

The mediating effect of affective balance on the relationship between childhood maltreatment and suicidal ideation was tested using PROCESS Model 4. As presented in Table 2, childhood maltreatment significantly positively predicted suicidal ideation (*β* = 0.076, *t* = 17.26, *p* < 0.001, 95% CI [0.067, 0.085]). When both childhood maltreatment and affective balance were included in the regression model, childhood maltreatment continued to significantly predict suicidal ideation (*β* = 0.067, *t* = 15.29, *p* < 0.001, 95% CI [0.058, 0.075]), while affective balance significantly negatively predicted suicidal ideation (*β* = −0.298, *t* = −11.74, *p* < 0.001, 95% CI [−0.348, −0.248]). Bootstrap analysis revealed a significant indirect effect of childhood maltreatment on suicidal ideation through affective balance (*β* = 0.010, 95% CI [0.007, 0.012]). This finding supports Hypothesis 1, indicating that affective balance partially mediates the relationship between childhood maltreatment and suicidal ideation among female college students.

**Table 2:** Results of the mediation and moderated mediation analyses.

Dependent variable	Independent variable	*β*(SE)	*t*	95% CI	*R* ^2^
Suicidal ideation	Childhood maltreatment	0.067(0.004)	15.285***	[0.058 to 0.075]	0.116
	Affective balance	−0.298(0.025)	−11.741***	[−0.348 to 0.248]	
Affective balance	Childhood maltreatment	−0.035(0.004)	−9.816***	[−0.042 to 0.028]	0.047
	Expressive suppression	−0.004(0.009)	−0.440	[−0.021 to 0.013]	
	Childhood maltreatment × expressive suppression	0.003(0.001)	5.364***	[−0.002 to 0.005]	

*N* = 2,280. All continuous variables were standardized before the analysis. ***p < 0.001.

### 3.4 Moderating effect of expressive suppression

To test the moderating effect of expressive suppression on the relationship between childhood maltreatment and affective balance, PROCESS macro (Model 7) was used. As presented in Table 2, childhood maltreatment significantly negatively predicted affective balance (*β* = −0.035, *t* = −9.82, *p* < 0.001, 95% CI [−0.042, −0.028]), while the main effect of expressive suppression on affective balance was not significant (*β* = −0.004, *t* = −0.44, *p* > 0.05, 95% CI [−0.021, 0.013]).

Importantly, the interaction between childhood maltreatment and expressive suppression significantly predicted affective balance (*β* = 0.003, *t* = 5.36, *p* < 0.001, 95% CI [0.002, 0.005]). The significant interaction effect accounted for an additional 2.1% of the variance in affective balance (Δ*R*^2^ = 0.021, *p* < 0.001), supporting the moderating role of expressive suppression in the relationship between childhood maltreatment and affective balance.

**Figure 2:**
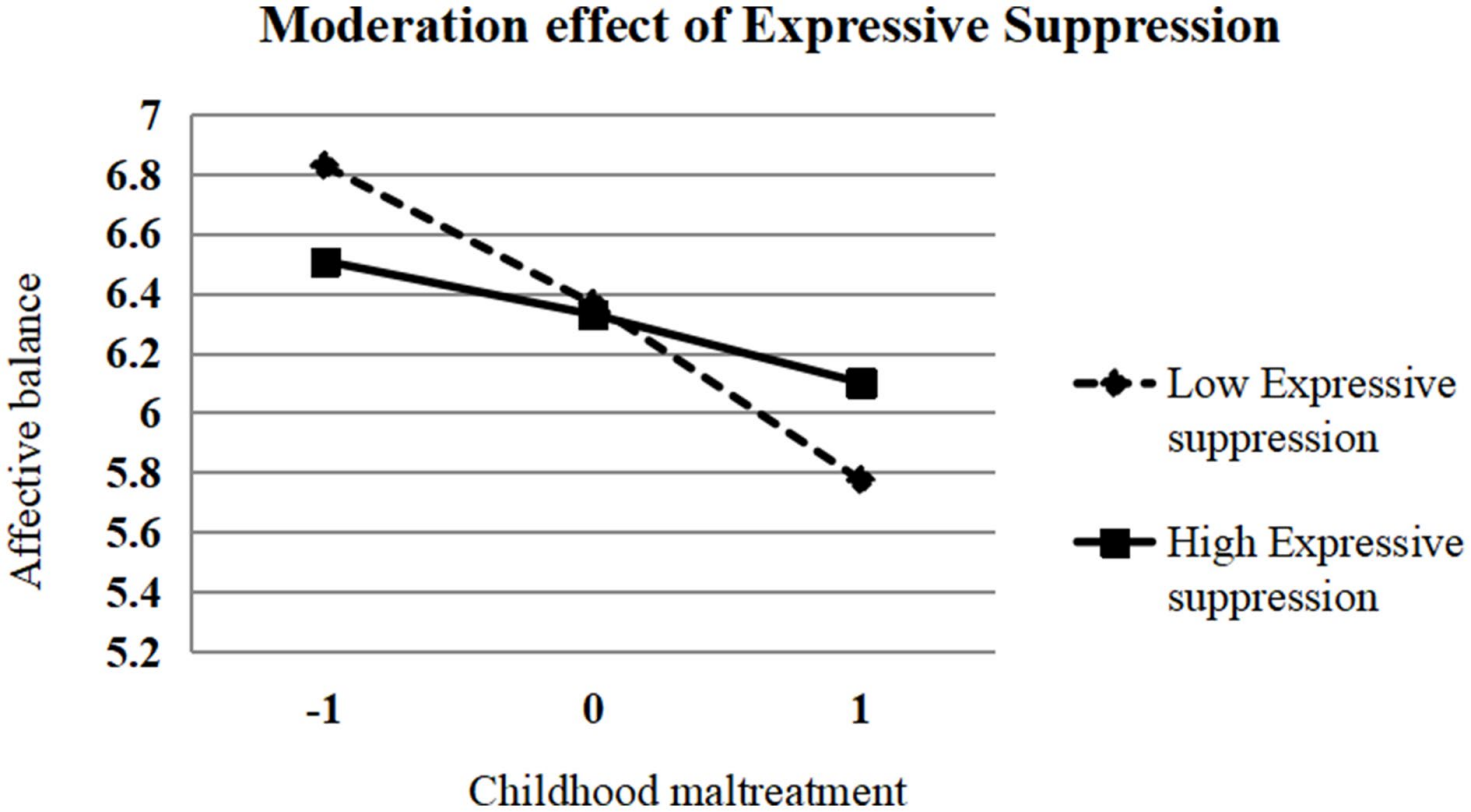
Moderating effect of expressive suppression on the relationship between childhood maltreatment and affective balance (lines represent simple slopes at ±1 SD from the mean of the moderator variable).

The simple slope analyses (Figure 2) revealed that when expressive suppression was low (−1 SD), the negative effect of childhood maltreatment on affective balance was stronger (*β* = −0.051, *SE* = 0.005, *t* = −10.21, *p* < 0.001, 95% CI [−0.060, −0.041]), whereas when expressive suppression was high (+1 SD), the negative effect of childhood maltreatment on affective balance was weaker, although still significant (*β* = −0.020, SE = 0.004, *t* = −4.67, *p* < 0.001, 95% CI [−0.028, −0.011]). These findings suggest that high levels of expressive suppression attenuate the negative impact of childhood maltreatment on affective balance among female college students, consistent with the direction anticipated in Hypothesis 2. Although statistically significant, the effect sizes are relatively small, indicating that expressive suppression plays a modest protective role in buffering the association between childhood maltreatment and affective balance.

### 3.5 Moderated mediation analysis

The index of moderated mediation was significant (*β* = −0.001, 95% CI [−0.001, −0.000]), indicating that the indirect effect of childhood maltreatment on suicidal ideation through affective balance was moderated by expressive suppression. In particular, the indirect effect was stronger at low levels of expressive suppression (*β* = 0.015, 95% CI [0.011, 0.020]) compared with high levels of expressive suppression (*β* = 0.006, 95% CI [0.003, 0.009]). These results suggest that expressive suppression serves as a protective factor that buffers the indirect effects of childhood maltreatment on suicidal ideation through affective balance.

## 4 Discussion

### 4.1 Summary of the findings

This study investigated how childhood maltreatment contributes to suicidal ideation among Chinese female college students through emotional and cultural mechanisms. Consistent with the hypothesized model, affective balance mediated the relationship between childhood maltreatment and suicidal ideation, whereas expressive suppression moderated this relationship. Higher levels of childhood maltreatment have been associated with reduced affective balance, reflecting elevated negative and diminished positive affect, which predicts greater suicidal ideation. However, this indirect association was significantly weaker among individuals with higher levels of expressive suppression, suggesting that suppression buffers the emotional consequences of early adversity. These results support both proposed hypotheses and validate the culturally moderated functional hypothesis of expressive suppression.

### 4.2 Affective balance as a mediating mechanism

The mediating role of affective balance aligns closely with the dual-process model of affect (Diener et al., 2018), which posits that subjective wellbeing and psychopathology hinge on the dynamic interplay between positive and negative affect. Consistent with previous research (Matud et al., 2022), emotional equilibrium serves as a central determinant of mental health and a proximal mechanism of suicidal cognition. Previous studies have indicated that disruptions in affective balance are predictive of depression and suicidal ideation across cultural groups (Humphreys et al., 2020; Veilleux et al., 2020; Chen et al., 2021).

Childhood maltreatment has consistently been associated with heightened negative affectivity and impaired emotional regulation capacity (Teicher and Samson, 2016; Humphreys et al., 2020). These experiences alter stress reactivity and emotional appraisal processes, leading to chronic emotional dysregulation. The present findings corroborate previous evidence that early adversity reduces the ability to sustain positive affect and amplifies negative emotional reactivity (Goncharenko et al., 2021). The resultant affective imbalance may, in turn, generate cognitive vulnerability to suicidality by fostering hopelessness, rumination, and self-blame (Brausch et al., 2025; da Silva et al., 2024; Martin et al., 2023).

The current study extends these findings to a collectivistic cultural context and a female college population, highlighting affective balance as a transdiagnostic emotional mediator through which early adversity translates into psychological distress. This underscores the importance of targeting the affective equilibrium in suicide prevention efforts, particularly among adolescents and young adults with a history of maltreatment. However, it is crucial to note that our findings are based on a sample drawn from eight provinces in China and might not fully capture the diversity of emotion regulation practices across all Chinese sub-groups.

### 4.3 Expressive suppression as a culturally adaptive moderator

The central contribution of this study is the moderating role of expressive suppression. Although Western literature commonly characterizes expressive suppression as maladaptive and links it to reduced interpersonal satisfaction and heightened physiological arousal (Gross and John, 2003; Butler et al., 2007), cross-cultural research increasingly challenges the universality of this interpretation. Studies among East Asian populations have determined that suppression is not uniformly detrimental; instead, it may yield neutral or even beneficial outcomes when aligned with prevailing cultural norms (José et al., 2011).

Our results corroborate this culturally nuanced perspective. Among Chinese female students, expressive suppression mitigated the negative emotional effects of childhood maltreatment on affective balance, implying that suppression serves as a buffer rather than a harmful effect. This finding supports the cultural fit hypothesis (Friedman et al., 2010), which asserts that the adaptiveness of emotion regulation strategies depends on their congruence with cultural expectations. Specifically, our findings provide empirical support for the Culturally Moderated Function Hypothesis, which posits that the psychological functions and outcomes of emotion regulation strategies are fundamentally shaped by cultural context, with the same strategy serving different purposes and having different consequences across cultural settings.

In the collectivistic Chinese context, emotional restraint and social harmony are strongly endorsed (José et al., 2011; Cheng et al., 2009). Suppressing overt negative emotions is socially reinforced as a sign of relational attunement and maturity rather than avoidance. Empirical evidence further suggests that habitual suppression among East Asians does not necessarily impair social functioning or psychological wellbeing (Su et al., 2015). Emotion suppression in such contexts serves interpersonal goals such as maintaining peaceful relationships and preventing social disruption (José et al., 2011). Expressive suppression can act as a culturally sanctioned self-regulatory mechanism that stabilizes affective processes and maintains psychological and social equilibrium following stress or trauma.

### 4.4 Theoretical integration with other cultural models of emotion

In addition to the cultural fit hypothesis, our findings are also consistent with other cultural models of emotion. From a dialectical emotion regulation perspective (Mauss and Butler, 2010), East Asian cultural contexts often tolerate—indeed, expect—the coexistence of positive and negative affect and promote flexibility in managing emotional contradiction rather than maximizing hedonic positivity. In this framework, expressive suppression may function less as avoidance and more as a context-sensitive strategy for keeping emotional arousal and its social consequences in balance, thereby weakening the extent to which childhood maltreatment disrupts affective equilibrium. Likewise, the present pattern aligns with cultural models that distinguish socially engaging versus socially disengaging emotions (Kitayama et al., 2006). In interdependent settings, emotions and their regulation are closely tied to relationship maintenance; regulating expression can support socially engaging goals (e.g., preserving harmony, preventing burdening others) even when internal distress is present. Thus, the buffering role of expressive suppression observed here may reflect its interpersonal function in promoting relational coordination, which indirectly helps sustain affective balance and reduces suicidal ideation risk. Integrating these perspectives, expressive suppression in the current sample may be understood as a culturally shaped, socially oriented regulation tactic that supports both intrapersonal affective stability and interpersonal harmony.

### 4.5 An integrated theoretical model

By synthesizing these findings, this study advances an integrated theoretical model that links developmental adversity, affective mechanisms, and culturally grounded emotion regulation. The model posits the following:

1. Childhood maltreatment is a distal risk factor that disturbs core affective systems.
2. Affective balance serves as a proximal emotional mechanism through which disturbances manifest as suicidal cognition.
3. Expressive suppression, shaped by the cultural values of restraint and harmony, moderates this mechanism by either buffering or amplifying emotional disequilibrium, depending on cultural congruence.

This model extends Gross and John’s (2003) process model of emotion regulation by incorporating cultural contingencies into the framework. While the original model conceptualizes regulation strategies as universally adaptive or maladaptive based on their cognitive–emotional consequences, the revised model situates their efficacy within sociocultural ecologies. In collectivistic cultures, suppression fits the normative expectations of modesty, composure, and socially sensitive self-control (Brausch et al., 2025; da Silva et al., 2024; Martin et al., 2023), thereby enhancing its adaptive potential. Conversely, in individualistic contexts that emphasize authenticity and emotional transparency, suppression may lead to social incongruence and psychological strain (Butler et al., 2007).

Thus, this integration transcends the dichotomy between “adaptive” and “maladaptive” regulation, positioning cultural meaning as a critical moderator of emotional function. This contributes to the emerging framework of culturally contextualized emotion regulation, in which strategy effectiveness is derived not from intrinsic qualities but from its cultural fit within a socially constructed emotional environment.

### 4.6 Theoretical and practical implications

The proposed model advances the field in three substantial ways.

First, it offers a conceptual refinement by integrating developmental, emotional, and cultural processes into a coherent framework for understanding suicidality. This approach aligns with recent calls for context-sensitive emotion regulation models (Aldao, 2013).

Second, the model contributes to a theoretical recalibration of Gross and John’s (2003) paradigm by incorporating the cultural context as an essential determinant of regulatory efficacy. Third, it bridges cross-cultural psychology and affective science by demonstrating how emotional adaptiveness depends on the interactions between individual regulatory tendencies and shared cultural norms.

Third, the model provides brief recommendations for key stakeholders in collectivistic cultural settings (e.g., Chinese universities), with particular attention to trauma-exposed female students, as summarized in Table 3.

**Table 3:** Clinical implications: stakeholder-specific recommendations based on the present findings.

Stakeholder	Recommendations
Clinicians (counselors/psychotherapists)	(1) Assess *affective balance* (low positive affect plus elevated negative affect) as a proximal, modifiable indicator associated with suicidal ideation, particularly among individuals reporting childhood maltreatment. (2) Avoid uniformly pathologizing *expressive suppression*; evaluate its function (e.g., harmony maintenance vs. avoidance) and its costs/benefits for the client. (3) Promote *regulatory flexibility* by pairing culturally normative restraint with complementary skills to restore affective balance (e.g., mindfulness-based emotion awareness, behavioral activation/positive activity scheduling, self-compassion, and graded, context-appropriate expression). (4) When maltreatment history and low affective balance co-occur, implement stepped *suicide risk screening*, safety planning, and referral pathways within campus mental health services.
Educators (faculty/advisors/student affairs)	(1) Strengthen early identification by training staff to recognize persistent low positive affect, withdrawal, and hopelessness as possible warning signs and to use culturally sensitive referral language. (2) Create low-stigma help-seeking channels (e.g., anonymous screening, brief drop-in consultations, peer gatekeeper programs) that reduce barriers for students who habitually inhibit emotional expression. (3) Support affective balance in daily campus life through structured, relationship-safe activities (e.g., belonging interventions, mentoring, group-based skill workshops) that increase positive affect without forcing public disclosure.
Policymakers (university and public health decision-makers)	(1) Embed *trauma-informed care* in campus systems, including routine screening protocols, clear crisis response procedures, and supervision/training standards for counselors working with suicide risk. (2) Resource multi-tier prevention (universal emotional well-being programs, indicated interventions for trauma-exposed students, and rapid access for high-risk cases) rather than relying solely on individual counseling. (3) Culturally adapt intervention guidelines by framing emotion regulation goals around *balance and relational functioning* (not only emotional expression) and evaluating programs using both wellbeing and suicide-risk indicators.

### 4.7 Limitations and future directions

This study has several limitations that warrant consideration. First, the cross-sectional design precludes definitive causal inferences regarding the temporal ordering of childhood maltreatment, affective balance, and suicidal ideation. Longitudinal or prospective designs would help clarify the developmental trajectories and dynamic processes underlying these associations.

Second, all variables were assessed using self-report measures, which may be vulnerable to recall bias and social desirability effects. These concerns are particularly salient when assessing emotionally sensitive experiences, such as childhood adversity and emotional inhibition. Future research should adopt multi-method approaches—such as behavioral tasks and informant reports—to mitigate these biases. In addition, incorporating behavioral and/or physiological indexes of emotion regulation would complement self-report data and provide a more comprehensive assessment.

Third, the internal consistency of the Bradburn Affect Balance Scale in the present sample was modest (Cronbach’s *α* = 0.61); this level is below the conventional threshold for satisfactory reliability, although it is comparable to values reported in prior studies using this measure in Chinese populations (Wan et al., 2017). As noted in the Methods section, this scale was selected for its theoretical alignment with the dual-process model of affect and its established use in cross-cultural research. While alternative measures, such as the PANAS, were considered, the Bradburn scale’s direct operationalization of affective balance as a single dimension better suited our theoretical framework. Future studies may benefit from employing affect measures with higher internal consistency or modeling affective balance as a latent construct to more explicitly account for measurement error. From a psychometric standpoint, reduced reliability in a mediating variable introduces random measurement error, which typically attenuates regression coefficients and biases effect estimates toward zero. Consequently, the indirect effect of childhood maltreatment on suicidal ideation through affective balance observed in this study is likely to represent a conservative estimate of the true mediation effect rather than an overestimation. Importantly, despite this limitation, the mediation pathway remained statistically significant with narrow bootstrap confidence intervals, underscoring the robustness of the emotional mechanism identified.

Fourth, the sample was drawn from eight provinces in China, which might not fully represent the country’s diverse sociocultural landscape. The research findings might not be generalizable to all sub-groups within China (e.g., rural vs. urban populations, distinct ethnic communities). China has significant regional, ethnic, and socioeconomic diversity, which could influence emotion regulation practices. Thus, our findings should be interpreted with caution regarding their applicability across all Chinese cultural contexts. Future research should incorporate measures of regional or subcultural norms to enhance conceptual precision. This could include assessments of local cultural norms, urban–rural distinctions, ethnic identity, and regional values. Such approaches would provide more nuanced insights into how emotion regulation strategies function across different sociocultural contexts within the same national boundary.

Finally, the sample consisted exclusively of Chinese female college students, which limits the generalizability of the findings to male, non-college populations, or individuals from different cultural backgrounds. Gendered emotional norms and sociocultural expectations may shape the function of expressive suppression differently across groups. Future research should examine the proposed moderated mediation model in more diverse samples and across cultural contexts, including direct cross-cultural comparisons.

## 5 Conclusion

This study sheds light on how childhood maltreatment exerts both universal and culture-specific effects on suicidal ideation through the mediating role of affective balance and the moderating function of expressive suppression. Extending beyond Western-centric models, the findings revealed that expressive suppression, which is often perceived as maladaptive, can serve contextually adaptive purposes in collectivistic cultures that emphasize harmony and restraint. The proposed culturally embedded theoretical model expands Gross and John’s (2003) emotion regulation theory and promotes a more inclusive understanding of how culture shapes emotional function and psychological resilience. By situating emotion within the cultural ecology, this study contributes to the development of a truly global science of emotion and mental health.

## Data Availability

The raw data supporting the conclusions of this article will be made available by the authors, without undue reservation.
